# The complete genome, comparative and functional analysis of *Stenotrophomonas maltophilia *reveals an organism heavily shielded by drug resistance determinants

**DOI:** 10.1186/gb-2008-9-4-r74

**Published:** 2008-04-17

**Authors:** Lisa C Crossman, Virginia C Gould, J Maxwell Dow, Georgios S Vernikos, Aki Okazaki, Mohammed Sebaihia, David Saunders, Claire Arrowsmith, Tim Carver, Nicholas Peters, Ellen Adlem, Arnaud Kerhornou, Angela Lord, Lee Murphy, Katharine Seeger, Robert Squares, Simon Rutter, Michael A Quail, Mari-Adele Rajandream, David Harris, Carol Churcher, Stephen D Bentley, Julian Parkhill, Nicholas R Thomson, Matthew B Avison

**Affiliations:** 1 Pathogen Sequencing Unit, The Wellcome Trust Sanger Institute, Hinxton, Cambridge, CB10 1SA, UK; 2 Department of Cellular and Molecular Medicine, University of Bristol, School of Medical Sciences, University Walk, Bristol, BS8 1TD, UK; 3 Biomerit Research Centre, Department of Microbiology, Biosciences Institute, National University of Ireland, Cork, Ireland

## Abstract

**Background:**

*Stenotrophomonas maltophilia *is a nosocomial opportunistic pathogen of the Xanthomonadaceae. The organism has been isolated from both clinical and soil environments in addition to the sputum of cystic fibrosis patients and the immunocompromised. Whilst relatively distant phylogenetically, the closest sequenced relatives of *S. maltophilia *are the plant pathogenic xanthomonads.

**Results:**

The genome of the bacteremia-associated isolate *S. maltophilia *K279a is 4,851,126 bp and of high G+C content. The sequence reveals an organism with a remarkable capacity for drug and heavy metal resistance. In addition to a number of genes conferring resistance to antimicrobial drugs of different classes via alternative mechanisms, nine resistance-nodulation-division (RND)-type putative antimicrobial efflux systems are present. Functional genomic analysis confirms a role in drug resistance for several of the novel RND efflux pumps. *S. maltophilia *possesses potentially mobile regions of DNA and encodes a number of pili and fimbriae likely to be involved in adhesion and biofilm formation that may also contribute to increased antimicrobial drug resistance.

**Conclusion:**

The panoply of antimicrobial drug resistance genes and mobile genetic elements found suggests that the organism can act as a reservoir of antimicrobial drug resistance determinants in a clinical environment, which is an issue of considerable concern.

## Abstract 2

The complete *Stenotrophomonas maltophilia* genome sequence suggests that it can act as a reservoir of antibiotic resistance determinants.

## Background

The rise of antimicrobial drug resistance in bacteria is one of the biggest threats to healthcare provision in the developed world. Few new antimicrobial drugs are undergoing clinical trials, and almost none are effective against Gram-negative multi-drug resistant (MDR) pathogens [1]. A return to the pre-antibiotic era is a possibility, and for some infections is the current reality [2].

Antimicrobial resistance in historically common pathogens is usually either acquired on a mobile genetic element or results from a mutation [3]. However, some opportunistic pathogens are intrinsically resistant to the actions of a number of antimicrobial classes. These tend to be of environmental origin, and their intrinsic drug resistance determinants either provide resistance to antibiotics produced by competitors, or represent broad-spectrum methods for removing toxic compounds or waste products that, by chance, protect against antimicrobials [3,4]. It is known that established opportunistic infections are very difficult to treat due to the MDR nature of the causative bacteria [5].

The most common intrinsically MDR opportunistic pathogens are the non-fermenting Gram-negative bacilli typified by *Pseudomonas aeruginosa*. In this case, intrinsic resistance is due to a battery of efflux pumps, specific antibiotic hydrolyzing enzymes, and intrinsically low outer membrane permeability. When intrinsically MDR bacteria then acquire resistance to those few drugs that can kill them, the result is an isolate resistant to all clinically available antimicrobials. This pan-resistant phenotype is observed in *P. aeruginosa *isolates with increasing frequency [6].

*S. maltophilia *is the third most common nosocomial non-fermenting Gram-negative bacilli [7]. A recent study of intensive care patients in the USA found that 4.3% of almost 75,000 Gram-negative infections studied were caused by *S. maltophilia *[8]. Isolates are intrinsically resistant to β-lactams, aminoglycosides, macrolides, and many older quinolones [7].

*S. maltophilia *is found in soil and water, and routinely resides in showerheads and other moist places where it grows as biofilm. It is a truly opportunistic pathogen, and patient to patient spread has not been reported, though small outbreaks have been seen due to contaminated water sources [9]. Consistent with this, we find that isolates are generally genotypically and phenotypically diverse [10-12]. However, there is phylogenetic clustering, with about half of clinical isolates being very similar to each other, even across a wide geographical range. Members of this group, termed phylogenetic group A, may be better at causing infections than other *S. maltophilia *isolates [13]. The two most common diseases caused by *S. maltophilia *are bacteremia and pneumonia with infection being via an indwelling catheter or ventilator, respectively [9]. Respiratory tract colonization is seen in about a third of all cystic fibrosis (CF) patients; nevertheless, there is controversy as to whether this leads to a poorer clinical outcome or morbidity [14,15].

Bioinformatic and functional genomic analyses on the complete genome sequence emphasize factors with proven or potential contribution to antibiotic resistance, persistence and virulence. The findings reveal the remarkable capacity of *S. maltophilia *for multidrug resistance and environmental adaptability that underpins its importance as an emerging opportunistic nosocomial pathogen.

## Results and discussion

### Total genome overview

The sequenced isolate is from a typical presentation: an elderly male patient undergoing chemotherapy at the Bristol Oncology Unit, Bristol, UK in 1998 developed a bloodstream infection that did not respond to therapy with piperacillin/tazobactam, ceftazidime or imipenem. *S. maltophilia *K279a was cultured from a blood sample taken shortly before death [16]. K279a falls into phylogenetic group A, and has typical antimicrobial resistance properties [13,17,18]. Accordingly, it was thought suitable as a representative genome sequence strain.

**Figure 1:**
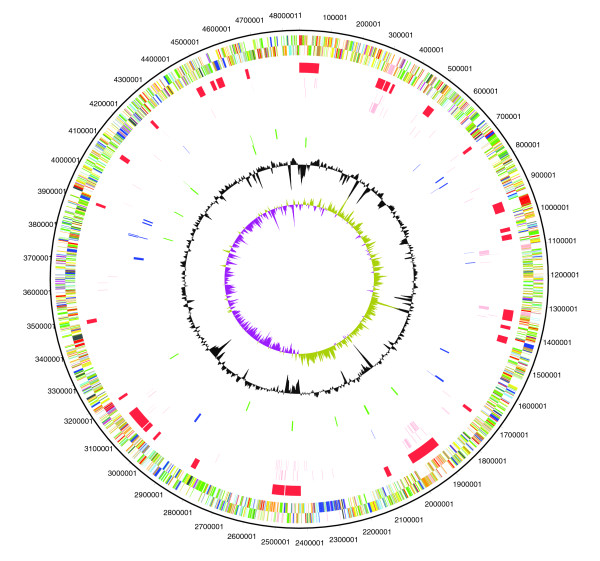
Circular diagram of the main features of K279a. The circles show (outermost to innermost): 1, DNA coordinates (black); 2, color coded annotation (the CDSs are color coded according to function: blue = pathogenicity/adaptation; dark grey = essential metabolism; red = DNA replication/transcription/restriction-modification; green = transmembrane/outer membrane; cyan and magenta = degradation of large and small molecules, respectively; yellow = intermediary metabolism; light green = hypothetical; light blue = regulators; orange = conserved hypothetical; brown = pseudogenes; pink = transposons and phage); 3, laterally transferred regions (determined by Alien Hunter with a cut-off score of 15); 4, transposons and phage (pink); 5, pili and fimbriae (blue); 6, RND efflux transporters (green); 7, GC skew; 8, GC deviation.

The genome consists of a single circular chromosome; no plasmids were detected (Figure 1). The total size is 4,851,126 bp with a G+C content of 66.7% G+C. Four copies of the rRNA operon and 74 tRNAs are present. These data have been submitted to EMBL under accession number AM743169.

### Drug resistance

In Gram-negative nosocomial pathogens, MDR is usually mediated by the over-production of resistance-nodulation-division (RND) type efflux pumps. These pumps tend to have broad substrate profiles, including organic solvents, disinfectants and antimicrobial drugs from a number of different classes. Cytoplasmic efflux is driven by dissipation of the proton-motive force across the inner membrane. Two additional components are needed to remove substrates from the cell, forming a tripartite efflux pump complex that spans the envelope. A particular periplasm-spanning membrane-fusion protein (MFP) is usually specific to each RND efflux protein, and it is common to find the pair encoded as part of an operon. A third component, the outer membrane protein (OMP), can be encoded in the same operon, but there tend to be fewer different OMPs than RND/MFP pairs in a cell, meaning that the OMPs are often promiscuous [19].

The K279a sequence carries nine RND-type efflux pump genes that fall into the drug resistance type based on sequence homology. Homologues of two known *S. maltophilia *tripartite efflux pump operons are present, *smeABC *(Smlt4474-6) and *smeDEF *(Smlt4070-2), representing MFP, RND and OMP genes, respectively, in each case. SmeABC was first characterized in the clinical *S. maltophilia *isolate ULA511 [20], which is phylogenetically closely related to K279a [21]. Disruption of *smeAB *in ULA-511, or in a hyper-resistant mutant background, had no effect on drug resistance. However, disruption of *smeC *reduced the minimum inhibitory concentration (MIC) of a variety of antimicrobials against ULA-511, so SmeC may act as an OMP in at least one functional tripartite antimicrobial efflux pump [20].

SmeDEF is over-produced in a hyper-resistant mutant of the clinical isolate *S. maltophilia *D457 [22], which is phylogenetically quite distinct to K279a [21]. SmeDEF over-expression causes hyper-resistance to fluoroquinolones, chloramphenicol and tetracycline in K279a [17]. Hyper-expression occurs either through loss-of function mutations in the locally encoded TetR-type transcriptional repressor, *smeT *[17,23], or through undefined mutations, which may affect another regulator of the concentration of the activator [17,24]. Characteristics of the nine *S. maltophilia *RND efflux pumps are described in more detail in Table 1.

**Table 1:** Characteristics of Sme efflux transporters in *S. maltophilia *K279a

Systematic ID	Name	Known or putative regulation mechanism	Closest match to a known antimicrobial efflux protein
Smlt4474-4476	SmeABC	Two component regulator (SmeSR, Smlt4477-8)	*S. maltophilia *SmeABC [20]
Smlt4070-4072	SmeDEF	Tet-R type (SmeT, Smlt4073)	*S. maltophilia *SmeDEF [17,22]
Smlt1829-1833	SmeVWX	LysR type (Smlt1827)	51%, 56% and 48% amino acid identity, respectively, to *P. aeruginosa *MexEF-OprN [64]
Smlt2201-2202	SmeYZ	Two component regulator (Smlt2199-30)	44% and 59% amino acid identity, respectively, to AdeAB of *A. baumanii *[65]
Smlt3170-3171	SmeGH	TetR type (Smlt3169)	39% and 49% amino acid identity, respectively, to AcrAB of *M. morganii *[66]
Smlt3788-3787	SmeMN	?	<30% identity to other known antimicrobial efflux proteins
Smlt3925-3924	SmeOP	TetR type (Smlt3926)	<30% identity to other known antimicrobial efflux proteins
Smlt4279/4281	SmeIJK	?	41%, 50% and 44% amino acid identity, respectively, to MtdABC of *E. coli *[25]

To determine involvement of the seven novel RND efflux pumps in intrinsic antimicrobial drug resistance in K279a, their genes were disrupted by suicide gene replacement to cause a significant intragenic deletion and frameshift mutation. MICs of a variety of antimicrobials were determined against the mutants in comparison to wild-type K279a. From this experiment, we conclude that SmeZ, SmeJ and SmeK are involved in intrinsic antimicrobial drug resistance in K279a (Table 2). A *smeJK *double mutant behaved identically to the individual mutants, leading to the conclusion that, as with their homologues *mtdBC *in *Escherichia coli *[25], their products cannot work separately. Disruption of *smeZ *markedly affects only aminoglycoside MICs; disruption of *smeJ *and/or *smeK *has a more general but subtle effect on resistance, lowering MICs of some aminoglycosides, fluoroquinolones and tetracyclines, but none dramatically.

**Table 2:** MICs of a variety of antimicrobials against *S. maltophilia *K279a and derivatives lacking specific functional RND efflux pump genes

	Gent	Kan	Ami	Tob	Ery	Chor	Mero	Imi	Azt	Ctz	Pip	Tet	Min	Trim	Sul	Cipro	Levo	Nor
K279a	16	256	64	32	>1,024	6	32	256	256	8	64	16	0.25	32	64	2	4	32
*smeJ*	**8**	256	**32**	32	>1,024	6	32	256	512	8	64	**8**	**0.125**	32	64	**1**	4	32
*smeK*	**8**	256	**32**	32	>1,024	6	32	256	512	8	64	**8**	**0.125**	32	64	**1**	4	32
*smeJK*	**8**	256	**32**	32	>1,024	6	32	256	512	8	64	**8**	**0.125**	32	64	**1**	4	32
*smeZ*	**1**	**128**	**32**	**16**	>1,024	6	32	256	512	8	64	16	0.25	32	64	2	4	32

Gent, gentamicin, Kan, kanamycin; Ami, amikacin; Tob, tobramycin; Ery, erythromycin; Chor, chloramphenicol; Mero, meropenem; Imi, imipenem; Azt, aztreonam; Ctz, ceftazidime; Pip, piperacillin; Tet, tetracycline; Min, minocycline; Trim, trimpethoprim; Sul, sulphamethoxazole; Cipro, ciprofloxacin; Levo, levofloxacin; Nor, norfloxacin.

Entries in bold indicate changes of the MIC in the mutants compared to wild type K279a. The units used are in mg/L

Other known and putative antibiotic resistance genes in the genome specify resistance via a number of mechanisms to β-lactams, chloramphenicol, aminoglycosides, fluoroquinolones and macrolides (Table 3). Many of the resistance genes are located on small islands with no obvious mobile DNA features (determined by Alien Hunter [26]), and may not all be expressed. Experimentally determined antibiotic modifying enzymes produced by K279a are β-lactamases L1 and L2 specifying resistance to all clinically available β-lactams except the monobactams [27], and the aminoglycoside modifying enzymes APH 3'II and AAC 6'I that together confer resistance to all clinically available aminoglycosides except gentamicin [18]. It has been reported previously that the *S. maltophilia *L1 and L2 β-lactamases might be encoded on a large 'plasmid-like element', but this was not confirmed using pulse-field gel electrophoresis [27]. Given that there is no plasmid in *S. maltophilia *isolate K279a, and that L1 and L2 are not encoded on a region of the chromosome that resembles an integrated plasmid, it is likely that the result reported previously reflected chromosomal contamination of a plasmid preparation, giving a false PCR positive for the L1 and L2 genes.

**Table 3:** Putative and known antimicrobial drug and heavy metal resistance genes in the *S. maltophilia *K279a genome sequence

Substrate	Gene	Putative gene product
Aminoglycoside	Smlt0191	Putative aminoglycoside phosphotransferase
Aminoglycoside	Smlt1669	Putative aminoglycoside 2' N-acetyltransferase
Aminoglycoside	Smlt2120	Known aminoglycoside 3' phosphotransferase
Streptomycin	Smlt2336	Putative streptomycin 3" phosphotransferase/kinase
Aminoglycoside	Smlt3615	Known aminoglycoside 6'N acetyltransferase
Spectinomycin	Smlt2125/*spcN*	Putative spectinomycin phosphotransferase
Chloramphenicol	Smlt0620/*cat*	Putative chloramphenicol acetyltransferase
Fluoroquinolone	Smlt1071/*qnrB*	Putative quinolone resistance protein
Macrolides	Smlt0032	Putative MFS-type tripartite efflux transporter
Macrolides	Smlt1537-9	Putative ABC-type tripartite efflux transporter
Macrolides	Smlt2642-3	Putative ABC efflux transporter and MFP
Multidrug	Smlt1528-30/*emrA,emrB*	Putative MFS-type tripartite efflux transporter
Multidrug	Smlt1830-31;33/*smeVWX*	Putative RND-type tripartite efflux transporter
Multidrug	Smlt2201-2/*smeYZ*	Putative RND-type efflux protein and MFP
Multidrug	Smlt2796-8	Multidrug/fusaric acid resistance protein
Multidrug	Smlt3170-1/*smeGH*	Putative RND-type efflux protein and MFP
Multidrug	Smlt3787-78/*smeMN*	Putative RND-type efflux protein and MFP
Multidrug	Smlt3924-25/*smeOP*	Putative RND-type efflux protein and MFP
Multidrug	Smlt4072-74/*smeDEF*	Known RND-type tripartite efflux protein
Multidrug	Smlt4279-81/*smeJKL*	Putative RND-type tripartite efflux proteins and MFP
Multidrug	Smlt4474-76/*smeABC*	Known RND-type tripartite efflux protein
Beta-lactams	*bla*_L1_	Known Beta-lactamase - L1
Beta-lactams	*bla*_L2_	Known Beta-lactamase - L2
Kasuagamycin	*ksgA*	Putative kasuagamycin resistance protein
Organic solvents	*ostA*	Organic solvent tolerance protein
Peroxide	*ohrA*	Organic hydroperoxide
Mercury	*merRTPA*	Mercury resistance
Copper	*copLABMGCDF *Smlt2445/Smlt2443 *copL*2*A*2*B*2 *copCD*	Copper resistance
Arsenic	*arsRCHR*2*C*2*B *Smlt2420	Arsenic resistance

Genes encoding six other putative RND family tripartite efflux pumps are found in the K279a genome sequence. However, these are more closely related to cation/metal efflux pumps than to antimicrobial RND efflux pumps, and are designated *SmmABC *to *TUV*. K279a additionally encodes several alternative heavy metal resistance mechanisms that are associated with a complex mobile region of DNA. These include arsenic, mercury, and copper resistance. Alternative copper resistance genes are specified elsewhere in the genome. Heavy metal resistance (to cadmium via an efflux protein) has been described in *S. maltophilia *D457R [28].

DNA acquired by lateral gene transfer was identified using Alien Hunter [26]. Putative transposons, both conjugative and complex as well as insertion sequence (IS) elements were found in K279a. Throughout the genome there are seven intact copies of a single unique IS element related to IS*Xac3 *of *Xanthomonas campestris *pv *campestris *(*X. campestris*) 8004, and two pseudogenic copies. Additional IS elements present in the sequence include IS*Hne3*/IS*111A*-like and IS*Psy9*-like (Table 4). Intriguingly, a single putative streptomycin 3" phosphotransferase gene (Smlt2336) has inserted between genes *clpS *and *clpA *relative to the *X. campestris *genome. To one side of Smlt2336 is a set of 36 and 18 bp direct repeats, perhaps suggestive of a footprint of a mobile element that may have inserted then excised.

**Table 4:** Potential mobile regions and their major characteristics

Mobile region	Putative length, approx. (bp)	G+C content (%)	Putatively bounded by (repeat length, bp)	Major characteristics
Potential conjugative transposon	43,769	62.7	19	Hypotheticals, lipoproteins and an efflux protein cargo
Potential complex transposon insertion	97,538	61.9	18	Efflux transporters, mercury, arsenic and copper resistance, co-integrate resolution and integrases. May be a multiple insertion*
Potential complex transposon insertion	52,344	60.5	20	*Tra *genes and adhesins, DNA repair, conserved and unique hypotheticals. May be a multiple insertion. Carries IS elements and Tn*5044 *similarity
IS*Xac3*-like	1,157	65.1	ND	Seven intact copies and two pseudogenic copies
IS*Hne3*/IS*111A*-like	915	61.7	ND	Eleven intact copies
IS*psy9*	1,352	58.8	ND	Four intact copies
Phage cluster 1	118,000	63.7	ND	Putative pseudogenic phage. Putative IS insertion and tRNA located centrally
Phage cluster 2	37,992	63.2	ND	Putative intact phage

Mobile regions were determined using an approach that combined using the Alien Hunter program, repeat analysis and by-eye comparisons between K279a and the *X. campestris *genome sequence by ACT analysis. *CDS (Smlt2465) in this feature shares 72.5% identity with a previously characterized transposase, Tn*5044*, from a *Xanthomonas *spp. isolated from a heavy metal mine in Russia [67]. *Stenotrophomonas *was at that time classified as *Xanthomonas*. ND, not detected.

There is no evidence in K279a for a class one integron specifying sulfonamide resistance as has recently been seen in a number of *S. maltophilia *isolates [29], and K279a is sensitive to trimethoprim-sulphamethoxazole.

*S. maltophilia *harbor giant phage [30]; although potential prophages were identified in K279a, there is no evidence for giant lysogenic phage.

### Secretion systems and extracellular enzymes

Type I, II (*sec*), IV and V (autotransporter) as well as the twin arginine secretion systems genes are present in the K279a genome sequence. Surprisingly, there are no type III secretion genes in K279a. Type III secretion components are related to the flagella apparatus [31]. The flagella apparatus of *S. maltophilia *is highly conserved with the *X. campestris *system and there is no evidence to suggest that these components could function in type III secretion. Secreted extracellular enzyme genes were found in the genome. K279a encodes non-hemolytic phospholipase C (plcN1, Smlt1755) as well as enzymes from the phospholipase D family. Phospholipase cleaves phospholipids to fatty acids and is implicated in virulence due to its ability to degrade cell membranes. There is evidence that phospholipases contribute towards virulence in *Burkholderia pseudomallei *[32]. Other extracellular enzymes, including DNase, gelatinase, hemolysin, lipases, proteinase K and proteases, have been characterized and implicated in disease in *S. maltophilia *[33]. The major extracellular protease of K279a, StmPr1 (Smlt0861), has also been implicated as a virulence determinant [34].

### Pili, fimbriae and adhesins

*S. maltophilia *produces various pili/fimbriae that are implicated in adhesion and biofilm formation [35]. This type of aggregative behavior is likely to be associated with colonization of biotic and abiotic surfaces, evasion of the host immune response as well as increased drug resistance.

The Smf-1 fimbrial operon includes Smlt0706-Smlt0709. These 17 kDa subunit fimbriae mediate adherence, participate at early stages of biofilm formation [36] and can agglutinate red blood cells. Smf-1, seen as peritrichous semi-flexible fimbriae of 5-7 nm under electron microscopy, are produced at 37°C but not 18°C. Two distinct loci, Smlt1508-12 and Smlt0732-6, comprise further sets of putative pili/fimbrial genes that include fimbrial subunit and chaperone/usher proteins.

A TadE-like pili/fimbrial gene cluster is located at Smlt2867-Smlt2875. In *Actinobacillus*, bundled Flp pili are required for tight adherence and strongly attached biofilm on solid surfaces *in vitro*, which is likely to be required in oral cavity colonization and initiation of periodontal disease [37].

Type IV pili are implicated in adherence and autoaggregation in enteropathogenic *E. coli*. In some species they have been associated with twitching motility and biofilm formation (for example, the obligate plant pathogen *Xylella fastidiosa *and *P. aeruginosa*). Subunits and associated apparatus specifying the type IV pilus are scattered throughout the genome of K279a. K279a also carries a gene cluster that shares significant similarity with a locus specifying the giant cable pilus of *Burkholderia cenocepacia*. This pilus has been implicated in the pathogenicity of *B. cenocepacia *in CF patients [38]. However, not all pathogenic CF isolates of *B. cenocepacia *carry *cbl *genes; this can also be the case in other *Burkholderia *spp. [39]. Alternative potential adhesins are encoded in the genome, including an afimbrial adhesin and Hep-hag family adhesins.

In this bacteremia-associated isolate, K279a, there are three members of the YadA family of BuHA proteins that contain numerous Hep-Hag repeat domains [40]. Two hemagglutinin/hemolysin family proteins are present as pseudogenes. Hemolysin activator Smlt1389, and outer membrane surface filamentous heamagglutinin (FHA) Smlt1390 and Smlt4452 are present. Filamentous heamagglutinin is an important virulence factor in *Bordetella pertussis*, being involved in related adhesion and spread of bacteria through the respiratory tract [41].

### Intercellular and intracellular signaling

Quorum sensing (cell-cell signaling) is important in infection models of *P. aeruginosa*, and quorum-sensing signals that coordinate biofilm formation have been identified in CF sputum along with biofilm-like structures [42]. *S. maltophilia *also carries out cell-cell signaling; however, the *S. maltophilia *system does not employ the usual LuxIR regulators [43,44]. Instead, *S. maltophilia *uses the *Xanthomonas *and *Xylella *signaling system mediated by a diffusible signal molecule, DSF [45,46]. DSF activity has been detected in a number of strains of *S. maltophilia*, including K279a, and controls resistance to several antibiotics, aggregative and biofilm behavior and virulence in a nematode model [47]. The K279a proteome contains no *n*-acyl homoserine lactone (*N*-AHL) synthases of either the LuxI or LuxM type and no LuxS protein (implicated in autoinducer 2 synthesis in a wide range of bacteria). K279a does encode a single LuxR type regulator with an *N*-AHL autoinducer-binding domain. Such orphan LuxR-like proteins have been described in *Xanthomonas oryzae *pv *oryzae *(*X. oryzae*) [48] and *X. campestris *[49], which do not synthesize *N*-AHLs. These proteins may interact with a plant host component rather than bind *N*-AHLs.

DSF perception in *X. campestris *is linked to altered levels of the second messenger cyclic di-GMP through the action of the HD-GYP phosphodiesterase domain regulator RpfG [50]. Cyclic-di-GMP regulates a range of functions, including developmental transitions, adhesion, biofilm formation and virulence in diverse bacteria [51]. Cyclic-di-GMP levels are influenced by synthesis and degradation acted on by the protein domains GGDEF, EAL and HD-GYP. K279a encodes 33 proteins with a potential role in cyclic di-GMP turnover: 3 proteins with an EAL domain; 18 with a single GGDEF domain; 10 with GGDEF and EAL domains; and two HD-GYP domain proteins, including RpfG. Most of these proteins contain additional signal input domains, suggesting that their activities (and therefore cyclic di-GMP levels) are responsive to diverse environmental cues.

### Polysaccharides

Polysaccharides are integral components of the extracellular matrix of bacterial biofilms and may play a role in resistance of bacteria to antibiotics. In xanthomonads, the *gum *gene cluster specifies production of the exopolysaccharide xanthan that is important in biofilm formation as well as being a commercially important product. *X. fastidiosa *produces fastidian gum, a truncated xanthan that is encoded by a reduced *gum *gene cluster [52]. There are no *gum *gene cluster orthologues in K279a; hence, this strain does not produce either xanthan or a modified version. Additionally, K279a does not carry genes for cellulose production, nor the exopolysaccharide cepacian, produced by some strains of *B. cenocepacia*.

Gene products implicated in the formation of intermediates of lipopolysaccharides and exopolysaccharides have been identified in K279a. XanAB are involved in UDP-glucose and GDP-mannose biosynthesis whilst RmlAC are involved in the synthesis and interconversion of TDP-sugars. XanB shares significant homology with phosphomannose isomerase, a key enzyme in the biosynthesis of *P. aeruginosa *alginate. Alginate is a key polysaccharide and is upregulated in CF sputum isolates from patients that have been infected with *P. aeruginosa *over a considerable length of time. Mutations in *xanB *and *rmlAC *affect biofilm formation and twitching motility in *S. maltophilia *WR-C [53]. The *xanA *gene, also known as *spgM*, is a phosphoglucomutase that shares similarity with *P. aeruginosa algC *[54]. K279a also specifies an orthologue of alginate lyase (Smlt1473), which is intriguing, since in CF lungs, the organisms are likely to be in contact with alginate-producing *P. aeruginosa*.

### Comparing the genomes of *S. maltophilia *and *X. campestris *- two sides of the same coin?

**Figure 2:**
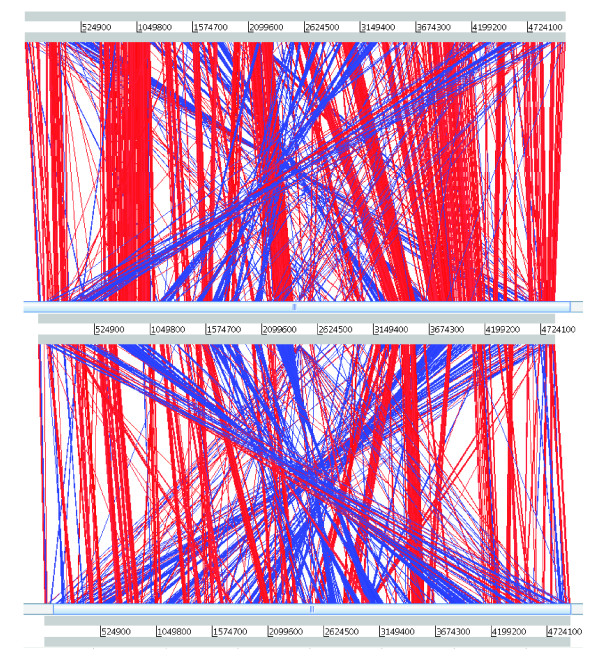
Artemis Comparison Tool (ACT) plot of K279a versus *X. campestris *and *X. oryzae*. The ACT plot against *X. campestris *8004 is shown at the top (NC_007086), *S. maltophilia *K279a is in the centre, and *X. oryzae *KACC10331 is at the bottom (NC_006834). Red bars denote matching regions, and blue bars denote inverted matching regions. The large number of genomic rearrangements can be seen.

**Figure 3:**
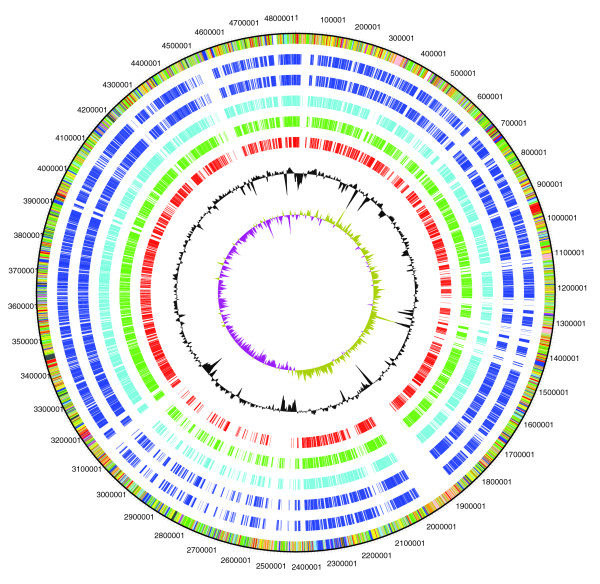
Circular diagram showing xanthomonad orthologues. Circles show (outermost to innermost): 1, DNA coordinates of the reference genome K279a; 2, color coded annotation file, all reading frames in the same circle; 3-7, orthologous genes determined by reciprocal best match analysis (3, *X. campestris *pv *campestris *8004 (NC_007086); 4, *X. campestris *pv *campestris *3391 (AE008922); 5, *X. campestris vesicatoria *(NC_007508); 6, *X. axonopodis citri *(NC_003919); 7, *X. oryzae *pv *oryzae *KACC10331 (NC_006834)); 8, GC skew; 9, GC deviation.

The K279a genome sequence was compared to that of *X. campestris *and *X. oryzae *using the Artemis Comparison Tool (ACT) (Figure 2). The extent of conserved regions between K279a and 8004 are difficult to visualize by ACT, mainly due to multiple chromosomal rearrangements. The first side of the 'coin' is illustrated by the use of K279a as a reference genome with a comparison of orthologous genes shared between K279a and sequenced xanthomonads on a circular genome representation. This comparison allows islands unique to K279a to be more clearly seen, the most obvious being the acquisition of a phage sited at 1,922,800 bp (Figure 3). Predicted functions of coding sequence (CDS) unique to K279a and those shared between 8004 and K279a are shown in Additional data file 1. Genes present in K279a that were not found in *X. campestris *may be applicable to human disease and are briefly described below.

**Figure 4:**
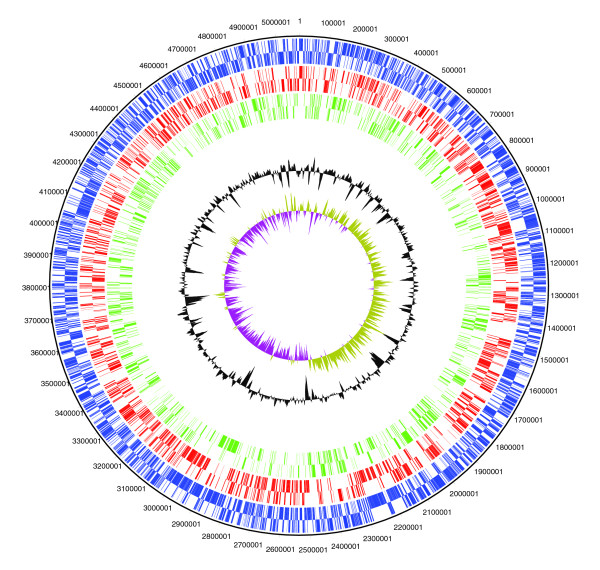
Circular diagram of orthologues shared between K279a and 8004. Circles show (outermost to innermost): 1, DNA coordinates of the reference genome 8004; 2, total CDS in both forward and reverse frames of the reference genome, *X. campestris *8004 (blue); 3, shared genes between 8004 and K279a (red); 4, genes unique to 8004 (green); 5, GC skew; 6, GC deviation. The *gum *gene cluster and type III secretion (*hrp/hrc*) cluster from *X. campestris *8004 can be seen clearly represented in the green (unique) circle at 2899664-2917444 and at 1424335-1427100, respectively.

The major regions of difference are phage and mobile elements; these encode both hypothetical and conserved hypotheticals as well as phage structural components (Table 3). In addition, several efflux transporter proteins in K279a are not present in 8004. Fimbrial/pili gene clusters are either divergent or not present in 8004. Other K279a acquisitions include hemagglutinins and hemolysins, two proteins with F5/8 type C coagulase domains, along with pseudogenes with hemagglutinin domains and similarities. Myosin cross-reactive antigen has also been acquired relative to *X. campestris*. Heavy metal resistance on a complex mobile element was acquired compared to 8004, as were some antibiotic resistance genes, especially those for aminoglycoside resistance. Although *S. maltophilia *is an obligate aerobe, the membrane-bound nitrate reductase that supports growth in the absence of oxygen with nitrate as a terminal electron acceptor is present in some strains [55]. The potential for microoxic growth is suggested in K279a, with the putative acquisition of formate dehydrogenase (*fdn*), the selenocysteine tRNA synthesis genes required for the Sel codon in *fdnG*, and the membrane-bound nitrate reductase (*nar*). Nar employs a molybdenum cofactor, and K279a *nar *genes cluster with Mo cofactor biosynthesis and transport genes, and a member of the FNR/CRP family of transcriptional regulators (*fnr2*, Smlt2767). An FNR homologue present in the K279a gene cluster suggests that the associated genes are only produced under limiting oxygenation since *E. coli *FNR regulates the aerobic-anaerobic switch [56]. Microarray analysis of *P. aeruginosa *under conditions encountered in CF lung (modeled by growth in CF lung sputum) indicates that *nar *gene expression is elevated [57]. Another FNR family member (Smlt2159) is located elsewhere. The potential for growth under microoxic conditions may enhance the pathogenicity of this organism, for example, by increasing its ability to grow in biofilm. K279a has gained some heat shock proteins that may be needed during pathogenic growth. Both genera share a high number of TonB dependent receptor proteins, a peculiarity of xanthomonads and epsilon proteobacteria [58]. Using *X. campestris *8004 as the reference genome in comparison to K279a, there are no large islands of acquisitions or losses. The flip side of the 'coin' is that genes present in *X. campestris *and absent in *S. maltophilia *are of relevance in plant disease (Figure 4). We can see the lack of the type III secretion system and *gum *gene cluster relative to *X. campestris*. Other plant pathogenic virulence determinants that are not present in K279a include the extracellular enzymes endoglucanase, polygalacturonate lyase, pectate lyase and cellulase. The *avr *genes involved in gene-for-gene resistance, such as *avr*BS1 [59], are also not present in K279a. Further studies of the genomic comparisons between *X. campestris *and *S. maltophilia *may reveal additional genes of medical interest or of interest in plant pathogenesis.

## Conclusion

The genome sequence of the bacteremia-associated *S. maltophilia *isolate K279a carries a startling array of antimicrobial drug resistance gene determinants. Knockout mutagenesis confirms the involvement of a number of novel RND efflux genes in resistance to a variety of different classes of antimicrobials.

The current drug of choice for treating *S. maltophilia *infections is trimethoprim-sulphamethoxazole, but resistance is seen in *S. maltophilia *isolates due to a mobile determinant [29,60]. Other drugs with reasonable activity against *S. maltophilia *are minocycline and newer fluoroquinolones [60]. However, mutants resistant to these last resort drugs are readily selected *in vitro*. One mutation may be sufficient to cause resistance to these drugs, and worryingly, this mutation can be selected for in the presence of a front-line antimicrobial such as amikacin [17].

The panoply of antimicrobial drug resistance genes and mobile genetic elements is an issue of clinical concern. *S. maltophilia *can also provide antibiotic resistance protection for sensitive *P. aeruginosa *and *Serratia marcescens *strains growing nearby [61]. Even more importantly, the organism potentially acts as a reservoir of antibiotic resistance determinants in medically relevant environments.

K279a possesses an unusual cell density-signaling pathway like that of its plant pathogenic xanthomonad relatives. K279a does produce extracellular enzymes such as protease StmPr1 and phospholipases; however, previous studies on clinical isolates have reported the production of other extracellular enzymes by *S. maltophilia*, suggesting that such virulence factors may be strain-specific. Comparison of K279a with *X. campestris *illustrates the movements of mobile genetic elements, the acquisition of potentially human pathogenic factors such as hemagglutinin, hemolysins and the loss of plant pathogenic factors such as the extracellular enzyme polygalacturonate lyase.

In conclusion, the *S. maltophilia *genome sequence reveals the capacity of this organism for environmental adaptations that presumably contribute to its persistence *in vivo*. As expected of a true opportunistic pathogen, the *S. maltophilia *genome does not suggest a highly virulent organism. However, the large number of pili/fimbrial genes does indicate a strong ability to attach to catheters and ventilators, from which infections of the blood or lungs arise. With its MDR phenotype and ability to attach, it is clear why this organism is persistent and difficult to eradicate. We are starting to build up a picture of an organism that is a true opportunist, which, while lacking many conventional key virulence determinants, has nevertheless emerged as a considerable threat.

## Materials and methods

### Sequencing strategy and annotation

*S. maltophilia *K279a was grown on Nutrient broth (Oxoid Cambridge, Cambridgeshire, UK) and genomic DNA was isolated using cetyltrimethylammonium bromide.

DNA was sonicated, size selected, and libraries were constructed in pUC19, pMAQ1b and pBACe3.6. The genome assembly was based on 3,381, 41,541 and 21,977 paired end-reads, respectively, from pUC19 libraries (of insert sizes 1.4-2.0 kb, 2.0-2.8 kb and 3.0-3.3 kb) and from 6,890, 314 and 69 paired end-reads, respectively, from pMAQ1b libraries (of insert sizes 5.5-6.0 kb, 9-10 kb and 10-12 kb), to give a 10.76-fold sequence coverage of the genome. We generated 1,250 and 106 reads, respectively, to produce a scaffold from 15-18 and 20-25 kb libraries in pBACe3.6. The genome was sequenced, finished and annotated as previously described [62]. To ensure that all bases were covered by reads on both strands or with different sequencing chemistries, and to fill gaps, 789 extra reads were generated. Repeats were bridged using read-pairs or end-sequenced PCR products. The total shotgun size was 53,580,262 Mb with a total genome coverage of 11.05-fold. Orthologous genes were determined by reciprocal best match analysis.

### Disruption of putative efflux pump genes and MIC determination

Genes were disrupted using a modified method of that previously described [17]. Genes were amplified by PCR in two non-overlapping fragments, with *Hin*dIII being introduced such that the two fragments could be ligated together, resulting in a mutant gene having a large deletion and a frameshift mutation. The primers used are listed in Additional data file 2. Mutated genes were used to replace wild-type sequences on the chromosome of K279a using the gene replacement approach described previously. Agar dilution MICs of antimicrobials against K279a and its derivatives were determined according to British Society for Antimicrobial Chemotherapy (BSAC)-approved methods [63].

## Abbreviations

ACT, Artemis Comparison Tool; BSAC, British Society for Antimicrobial Chemotherapy; CDS, coding sequence; CF, cystic fibrosis; IS, insertion sequence; MDR, multi-drug resistant; MFP, membrane fusion protein; MIC, minimum inhibitory concentration; *N*-AHL, *n*-acyl homoserine lactone; OMP, outer membrane protein; RND, resistance-nodulation-division.

## Authors' contributions

LCC, MBA, JMD and JP wrote the paper. GSV, AO, NP, AK, TC and EA provided DNA or analysis tools. VCG, DS, CA, and MAQ carried out experiments. LCC, VCG, JMD, GSV, MS, DS, AL, LM, KS, RS, SR, MAJ, DH, CC, SDB, JP, NRT and MBA analyzed data.

## Additional data files

The following additional data are available with the online version of this paper. Additional data file 1 shows the shared genes between K279a and *X. campestris*, and the genes unique to K279a determined by reciprocal best match analysis. Additional data file 2 is a table listing the primer sequences used in the generation of gene knock-outs.

## Supplementary Material

Additional data file 1 Shared genes between K279a and *X. campestris*, and the genes unique to K279a determined by reciprocal best match analysis. Click here for file

Additional data file 2 Primer sequences used in the generation of gene knock-outs. Click here for file
