# Supplementary material for: The complete genome, comparative and functional analysis of Stenotrophomonas maltophilia reveals an organism heavily shielded by drug resistance determinants
Source: Genome Biol. 2008 Apr 17;9(4):R74. doi: 10.1186/gb-2008-9-4-r74 (PMC2643945; doi:10.1186/gb-2008-9-4-r74)
Supplement: Additional data file 1 — Shared genes between K279a and X. campestris, and the genes unique to K279a determined by reciprocal best match analysis. [file gb-2008-9-4-r74-S1.doc]

**Additional Data File 1**

Genes shared between K279a and *X. campestris* 8004 and genes unique to K279a were determined following reciprocal best match analysis. The Monica Reilly classification scheme was used to separate the CDS into groups.

Hypothetical, no match CDS (0.0.0) comprised 11% of the unique genes. Conserved hypotheticals (0.0.2) were also represented more highly in genes unique to *S.maltophilia*, comprising 27%, and only 15.8% of the shared genes. Other key sections are the transmembrane proteins (4.1.1), making up 11% of the shared genes and 6.5% of the unique genes. Bacteriophage (5.1.4) comprised 3.7% of the unique genes and 0.13% of the shared genes, whilst IS elements and transposons (5.1.2) were also more highly represented in the unique genes, with 2.3% and 0.2%, respectively.
