# Supplementary material for: The complete genome, comparative and functional analysis of Stenotrophomonas maltophilia reveals an organism heavily shielded by drug resistance determinants
Source: Genome Biol. 2008 Apr 17;9(4):R74. doi: 10.1186/gb-2008-9-4-r74 (PMC2643945; doi:10.1186/gb-2008-9-4-r74)
Supplement: Additional data file 2 — Primer sequences used in the generation of gene knock-outs. [file gb-2008-9-4-r74-S2.doc]

**Additional Data File 2**

**Primers used in the disruption of putative RND-type efflux pump genes**.

| **Gene** | **5’ Proximal Primer Pair** | **3’ Proximal Primer Pair** |
| --- | --- | --- |
| ***smeH*** | 5’-GCAAGAACGTGAAGACCGATG-3’  5’-AAGCTTCGATGTTGGGATAGGACTC-3’ | 5’-AAGCTTGACATCGCCCAGGTG-3’  5’-CGACGGATCCGAGCCAAG-3’ |
| ***smeJ*** | 5’-GTCAACAATTACGCCGACTCG-3’  5’-AAGCTTGCGGTCGTTGAAGATCT-3’ | 5’-AAGCTTATGCGATCGTGATGAT-3’  5’-GTGCGCTGTTCCACTGC-3’ |
| ***smeK*** | 5’-GCGCGGCAACACCACTAC-3’  5’AAGCTTATGAACACCGCCACCAG-3’ | 5’-AAGCTTCCACCGTCTTCCTGTTC-3’  5’-GTTGACCACCACGCTGTACTG-3’ |
| ***smeN*** | 5’-CATGCACATCGATTTCCAGGTG-3’  5’-AAGCTTAGGCGCCGGTGTAGTC-3’ | 5’-AAGCTTCGTAGCTCAAGTGGAG-3’  5’-CGAAGATCTGCGTGCCC-3’ |
| ***smeP*** | 5’-TTGACCGGCAAGGGCTC-3’  5’-AAGCTTAGATGTTGCCGTAGGTG-3’ | 5’-AAGCTTGAGAAGGTGTGGATCGAG-3’  5’-GTCATCGCCAGCACCAGTG-3’ |
| ***smeW*** | 5’-CGATGACAAGGCCGTGCTGAC-3’  5’-AAGCTTAATCGTGATCAGGCCTG-3’ | 5’-AAGCTTCCATCAACGGCGTCGA-3’  5’-GGCCGACACCTGCACGTTC-3’ |
| ***smeZ*** | 5’-GGTGCTGGCGATCTTCAT-3’  5’-AAGCTTCCCAGTTCAACGCGGG-3’ | 5’-AAGCTTAGATTCCGTTCGACACC-3’  5’-CATGAAGTAGCCCTGGTCCTC-3’ |
